# Validation of the FASH (Functional Assessment Scale for Acute Hamstring Injuries) questionnaire for German-speaking football players

**DOI:** 10.1186/s13018-016-0464-0

**Published:** 2016-10-24

**Authors:** Heinz Lohrer, Tanja Nauck, Vasileios Korakakis, Nikos Malliaropoulos

**Affiliations:** 1 Institute for Sport and Sport Sciences, Albert-Ludwigs-Universität Freiburg i. Brsg, Schwarzwaldstraße 175, 79117 Freiburg, Germany; 2 European SportsCare Network (ESN), Zentrum für Sportorthopädie, Borsigstrasse 2, 65205 Wiesbaden-Nordenstadt, Germany; 3 European SportsCare, Harley Street 68, London, W1G 7HE UK; 4 Aspetar, Orthopaedic and Sports Medicine Hospital, Sport City Street, P.O. Box 29222, Doha, Qatar; 5 Institute for Postgraduate Studies in Manual Therapy, 111528 Athens, Greece; 6 Thessaloniki Sports and Exercise Medicine Clinic, Asklipiou 17, Thessaloniki, 54639 Greece; 7 National Track and Field Centre, Sports Medicine Clinic of S.E.G.A.S.,Kautatzoglion Stadion, Agiou Dimitriou 100, 54636 Thessaloniki, Greece; 8 Sports Clinic, Rheumatology Department, Barts Health NHS Trust, Bancroft Road, London, E1 4DG UK; 9 Centre for Sports and Exercise Medicine, Queen Mary University of London, Bancroft Road, London, E1 4DG UK

**Keywords:** Hamstring, Injuries, Disability, Validity testing, Questionnaire, FASH, Exercise testing

## Abstract

**Background:**

The FASH (Functional Assessment Scale for Acute Hamstring Injuries) questionnaire has been recently developed as a disease-specific self-administered questionnaire for use in Greek, English, and German languages. Its psychometric qualities (validity and reliability) were tested only in Greek-speaking patients mainly representing track and field athletes. As hamstring injuries represent the most common football injury, we tested the validity and reliability of the FASH-G (G = German version) questionnaire in German-speaking footballers suffering from acute hamstring injuries.

**Methods:**

The FASH-G questionnaire was tested for reliability and validity, in 16 footballers with hamstring injuries (patients’ group), 77 asymptomatic footballers (healthy group), and 19 field hockey players (at-risk group). Known-group validity was tested by comparing the total FASH-G scores of the injured and non-injured groups. Reliability of the FASH-G questionnaire was analysed in 18 asymptomatic footballers using the intra-class coefficient.

**Results:**

Known-group validity was demonstrated by significant differences between injured and non-injured participants (*p* < 0.001). The FASH-G exhibited very good test–retest reliability (intra-class correlation coefficient = 0.982, *p* < 0.001). Internal consistency was excellent (*α* = 0.938). Compared with the results presented in the original publication, no statistical differences were found between healthy athletes (*p* = 0.257), but patients’ groups and at-risk groups presented scoring differences (*p* = 0.040 and <0.001, respectively).

**Conclusions:**

The FASH-G is a valid and reliable instrument to assess and determine the severity of hamstring injuries in German footballers.

## Background

The “single most common injury subtype” in European professional football is hamstring injuries accounting for 12 % of all injuries [1]. This means that in this group 1.0 injury happens in 1000 h playing time. During the matches, the incidence is double [1]. In addition, hamstring injuries represent more than one third of all muscle injuries in football players [2, 3].

High-speed eccentric hamstring contractions are thought to indicate high-risk sports like football (soccer), American football, Australian football, rugby, and track and field [4]. Previous injuries are identified as the most important risk factor [5]. Therefore, rehabilitation plays a major role in injury prevention [6]. The effectiveness of interventions including eccentric training to prevent hamstring injuries is still under debate [7, 8].

To guide further respective research, the FASH (Functional Assessment Scale for Acute Hamstring Injuries) questionnaire has recently been developed as a disease-specific and self-administered questionnaire to grade the severity of symptoms (pain and function) in patients with hamstring injuries (Table 1) [4].

**Table 1:** FASH (Functional Assessment Scale for Acute Hamstring Injuries) and FASH-G (G = German version) items [4]

Questions	English version	German version
Q1	If you have had an acute hamstrings injury, please rate your current level of pain and/or, discomfort.	Beschreiben Sie bitte ihre jetzigen Schmerzen/Symptome, die nach der akuten hinteren Oberschenkelverletzung verblieben sind.
Q2	Are you currently taking part in your sport, training or, other physical activity?	Können Sie zur Zeit Sport ausüben oder sich körperlich belasten?
Q3	How much pain do you have during walking?	Wie viel Schmerz verspüren Sie während des Gehens?
Q4	How much pain do you have during jogging or, slow pace running?	Wie viel Schmerz verspüren Sie während des langsamen Laufens (Jogging)?
Q5	How much pain do you have during accelerating or, sprinting for 30 meters?	Wie viel Schmerz verspüren Sie beim Beschleunigen (Antreten)/oder beim Sprint über 30 m?
Q6	How much pain do you have during static stretching your hamstrings (toe touch in standing)?	Wie viel Schmerz verspüren Sie, wenn Sie ihre hintere Oberschenkelmuskulatur statisch dehnen (beim Versuch im Stehen mit gestreckten Knien die Fußzehen zu berühren)?
Q7	Do you have pain or, discomfort when performing a full weight-bearing lunge?	Wie viel Schmerz verspüren Sie beim dynamischen Stretching der hinteren Oberschenkelmuskulatur (Anheben des verletzten, gestreckten Beines)?
Q8	Do you have pain or, discomfort when performing a full weight-bearing lunge?	Verspüren Sie Schmerzen/ Symptome bei Ausführen eines tiefen Ausfallschrittes (verletztes Bein nach vorne stellen)?
Q9	Can you perform one Nordic exercise (partner exercise where you attempt to resist a forward-falling motion using your hamstrings throughout the whole range of motion to the ground)?	Können Sie mindestens eine Nordic-Übung durchführen (mit durch den Partner fixierten Unterschenkeln im Knien und mit geradem Rücken langsam nach vorne bis zum Boden beugen)?
Q10	Can you perform 3 one-legged jumps for distance?	Können Sie 3 einbeinige Weitsprünge mit dem verletzten Bein durchführen?

*Q* question

Even if developed de novo in Greek, English, and German languages, its validity and reliability were tested only in Greek-speaking patients mainly representing track and field athletes. “It is highly recommended that, after the translation and adaptation process, the investigators ensure that the new version has demonstrated the measurement properties needed for the intended application” [9].

The aim of this study is therefore to test the FASH tool to further prove its validity and reliability in a German-speaking football cohort.

## Methods

Following § 15 of the Hessian code of medical ethics (Berufsordnung Hessischer Ärztinnen und Ärzte), an ethics committee consideration is not needed for studies dealing with anonymised data. Respectively, the chairman of the local ethics committee considered the study to be not relevant for formal approval, because only anonymised data were evaluated in this study. Because of this anonymisation, the subjects and patients who were included in the study could not be identified from the obtained information and data. Therefore, consent to publish patient identifiable information and data was not necessary to be obtained. Verbal informed consent to participate was obtained from the participants, and the rights of the participants were protected.

### Participants

Sixteen footballers with hamstring injuries (patients’ group), 77 asymptomatic footballers (healthy group), and 19 hockey players (at-risk group) were recruited for the study (Table 2).

**Table 2:** Descriptive characteristics of the participants (median and range)

Groups	Age (years)	Height (cm)	Weight (kg)
Healthy (*n* = 77)	20 (18–44)	182 (162–196)^†^	74 (55–98)
At risk (*n* = 19)	23 (19–27)	180 (166–188)	80 (63–96)
Hamstring injuries (*n* = 16)	23 (18–31)	178 (164–183)^†^	75 (57–83)
Total (*n* = 112); 104 ♂, 8 ♀			

Values are presented as medians and ranges. The characteristics of the participants were gathered by the investigators during the first day of the assessment and before the administration of FASH

^†^Indicates statistically significant differences between groups, *p* < 0.05

The healthy football players represented two male teams from the 5th (*n* = 31), one team from the 6th (*n* = 10), and one from the 9th (*n* = 10) German football leagues, the male U 20 national team (*n* = 18), and a female team (*n* = 8) from the 1st German league. A first-league male field hockey team (*n* = 19) represented the at-risk control group. These participants were contacted via the trainers of the respective teams. The hamstring injury group consisted of 16 male football players from different leagues. These patients were recruited by their physiotherapists.

### Inclusion and exclusion criteria

Inclusion and exclusion criteria (Table 3) were mainly adapted from the FASH development study [4]. Participants were selected if they were 18 years or older and were competitively active in football or hockey. Participants of the control groups had to be part of one of the selected teams. The exclusion criteria were pregnancy and spinal symptoms. For the healthy groups, further exclusion criteria were pain and functional deficits in the hamstrings during physical activity. All these athletes had to be integrated in regular team practice and competition at the time the questionnaire was administered.

**Table 3:** Inclusion and exclusion criteria for the hamstring injury group

Inclusion criteria	Exclusion criteria
Acute injury	German not native language
Local tenderness on palpation at the injured site	Uncertain clinical diagnosis
Pain with resisted knee flexion	Verified or previously suspected posterior thigh muscle injury
Pain with resisted hip extension	Bilateral injuries
Pain with passive hip flexion with the knee extended	Extrinsic trauma to the posterior thigh
Provocation of pain on isometric contraction of posterior thigh muscles	Pain on palpation at the origin or insertion of the posterior thigh muscles
	Tendon avulsion or total rupture of any or all of the hamstring muscles
	Chronic low back pain and/or sciatica

Clinical examination followed detailed history taking. Athletes were included if all the inclusion criteria were present

For the hamstring injury group, patients were recruited from two sports physiotherapy centres. Football players who were diagnosed by a physician (history and physical examination) and were treated by their physiotherapists for acute hamstring injury were selected. All these athletes were not able to practice or compete at the time they filled out the questionnaire.

### Procedure

The FASH-G (G = German version) questionnaire was administered to all participants (*N* = 112). Different from the FASH development study [4], the participants filled out the questionnaire without the presence of an investigator. For the healthy and at-risk groups, respective team trainers were instructed by the authors to facilitate the application of the questionnaire. Instructed physiotherapists assisted the patients to complete the questionnaires. A healthy subgroup (*n* = 18) was chosen for the reliability analysis and completed the questionnaire twice within 48 to 60 h.

### Feasibility and acceptability

To appraise the acceptability and the ease of administration of the FASH-G, we subjectively analysed the filling-out process for problems with the questions.

### Statistical analyses

All statistical analyses were carried out using SPSS 22.0 (SPSS GmbH, Munich, Germany). Statistics were performed using descriptive data analysis as median and range and as mean with the respective standard deviation. The Kolmogorov–Smirnov test was applied to check out for normal distribution. Level of significance was set at *p* < 0.05.

#### Validity testing

Known-group validity and group differences were calculated using the Kruskal–Wallis test. Post hoc comparisons were performed using the Mann–Whitney *U* test, and Bonferroni corrections were applied for multiple comparisons. The internal consistency for the total FASH score was examined using Cronbach’s *α*. Intra‐class correlation coefficient (ICC) values >0.75 are considered as excellent, 0.75 to 0.40 as fair to poor, and <0.40 as poor [10].

#### Reproducibility testing

Reliability testing was performed by Spearman’s rank correlation test (rho). Test–retest reliability was defined by using two-way random‐effect ICC (type 2.1), because systematic differences are considered to be part of the measurement error [11]. The standard error of measurement (SEM; SEM = SD × √(1 − test–retest reliability coefficient)) and the minimal detectable change (MDC95; MDC = 1.96 × √2 × SEM) were additionally calculated [11–13].

#### Power analysis

The sample size required for the study was based on the ICC and the maximum width of the 95 % confidence intervals obtained from the development study of the original FASH questionnaire [14]. The formula used to calculate the sample size [15] was *n* = 16*p*(1 − *p*)/*w*2, where *p* is the expected ICC (selected ≥0.8) and *w* is the maximum width (0.40) of the 95 % confidence interval. The minimum total sample size per group was calculated to be 16. Despite that, the minimal sample size required ensuring the needs of internal consistency and stability testing is reported to be a minimum number of 100 participants [11, 16]. Due to the lack of availability of patients with hamstring injury during the study enrolment, we included more healthy individuals to fulfil the aforementioned criteria.

## Results

### Feasibility and acceptability

No problems with filling out the questionnaire were detected in the patients’ group. Even without additional communication with an investigator, these athletes were able to quickly go through and answer the questions. However, 25 % of the participants in the healthy control groups scored reduced values for questions 6, 7, 8, and 9. Additionally, question 1 produced some trouble for the uninjured athletes, because it is directly related to a hamstring injury.

### Validity

Validity testing (Table 4) revealed significant differences between the injured and all other tested groups (all *p* < 0.001).

**Table 4:** Total FASH scores for the groups in the study

Group	Number	Total FASH-G score	
		Median (range)	Mean (SD)
Healthy	77	100.0 (56–100)	97.5 (6.3)
At risk	19	90.0 (81–100)	90.1 (4.7)
Hamstring injuries	16	41.5 (1–88)	42.7 (29.9)

Data are presented as median and range

*SD* standard deviation

The scores for the individual FASH-G items within the group of hamstring-injured football players (Table 5) with one exception demonstrate a uniform result. Question 3 (“How much pain do you have during walking?”) is scored considerably higher when compared to all other item results.

**Table 5:** Individual FASH-G item results for the 16 footballers with hamstring injuries (patients’ group)

	FASH-G score	
FASH-G question	Median (range)	Mean (SD)
Q1	5.0 (0–10)†	5.1 (2.9)
Q2	5.0 (0–10)†	4.0 (3.7)
Q3	8.0 (1–10)	6.6 (3.4)
Q4	3.5 (0–10)	4.7 (4.0)
Q5	2.0 (0–8)	2.9 (3.1)
Q6	2.5 (0–10)	3.0 (3.3)
Q7	3.0 (0–10)	3.6 (3.2)
Q8	4.5 (0–10)	4.4 (3.5)
Q9	4.0 (0–10)	3.4 (3.4)
Q10	4.0 (0–10)	5.1 (4.3)

Numerical 0–10 rating for questions 1 and 3–8. Categorical rating system on an incremental range of values for questions 2, 9, and 10. 0 = worst result, 10 = best result

*SD* standard deviation

### Reliability

The 18 uninjured footballers scored median 100 (range = 63 to 100) in the initial and median 100 (range = 52 to 100) in the retest. Test–retest analysis revealed excellent temporal stability (*p* < 0.001; 95 % CI = 0.953–0.993). Spearman’s rho for test–retest reliability was *r* = 0.841 (*p* < 0.001). The standard error of measurement was 0.78, and the calculated minimal detectable change was 2.16. Internal consistency was excellent with a Cronbach *α* of 0.983 for the first and 0.917 for the second FASH-G assessment.

## Discussion

Based on the results of our study, the FASH-G questionnaire was proven to be a valid and reliable tool in evaluating the pain and functional status of German footballers with or without hamstring injury. The patients had no problems with filling out the questionnaire.

The FASH questionnaire was originally developed as a clinical tool to assess the severity and monitor hamstring injuries during rehabilitation. It is available in Greek, English, and German languages [4]. However, the validation process was performed only for the Greek FASH version, and the population used has overrepresented track and field athletes. Epidemiologic investigations demonstrate that hamstring injuries are not only the most frequent lesion in elite footballers but that its numbers are increasing in recent years [1, 2]. Therefore, we decided to subject the FASH-G questionnaire to further psychometric testing in a German football population.

Additional research has to be made to prove its worth to quantify the severity of hamstring injuries and for its use as an additional tool to guide rehabilitation. Only the clinical environment can determine return to sport decisions [17] even if MRI grading has been shown to correspond significantly with “lay-off” time [3]. In the present investigation, we only discriminated between acute hamstring injuries and hamstring-uninjured athletes. Therefore, in a next step, the question has to be solved if different types [18] of hamstring muscle lesions are mirrored by different FASH scores. Then, more objective return to play prognosis and decisions could be made by assessing the actual FASH score of an injured player.

Until now, the FASH is the only questionnaire for acute hamstring injuries. It was developed and cross-culturally adapted following a strict process [4]. In the generation process, three items (FASH questions 2, 3, and 4) of the VISA-H (Victorian Institute of Sport Assessment-Hamstring) questionnaire which evaluates proximal hamstring tendinopathy [19] were implemented in the FASH questionnaire. Concurrent validity has been demonstrated comparing these two instruments (*r* = 0.856; *p* < 0.01) [4].

While most items of the FASH questionnaire are unspecific and have already been used in different previously developed disease-specific questionnaires for the lower leg (VISA-A, VISA-P, FAAM), question 9 specifies an exercise which directly tests the hamstring muscles. This specific exercise has already demonstrated its worth to prevent hamstring injuries in soccer players [6].

As a result from our questionnaire evaluation process, we propose to have the FASH questionnaire filled out under the supervision of someone who is familiar with it. Specifically, our previously and currently uninjured participants faced difficulties in answering question 1 (“If you have had an acute hamstrings injury…”) and found no respective answering box to tick.

Another difficulty frequently arose with question 9, because the term “Nordic exercise” is not generally known in the German football population.

Individual FASH-G item analysis (Table 5) for the hamstring-injured footballers with the exception of question 3 demonstrated a uniform result. The higher value for question 3 (“How much pain do you have during walking?”) is most probable due to the fact that most hamstring injuries affect walking only for a few days and our questionnaire was administered later.

Compared with the uninjured male footballers of this study, the field hockey players scored significantly lower (*p* < 0.001, Table 4). We believe that the reason for this is given by the sensitivity of the questionnaire to other clinical presentations. At the time of the FASH assessment, the final of the season put the members of the first-league field hockey team under high pressure and overuse injuries not related to the hamstrings most probably caused this effect. This finding is in line with the known-group validity testing results during our original FASH validation [4]. Additionally, it underlines the fact that the FASH cannot be used as a diagnostic tool.

The male footballers were collected from different leagues, and the highest scores were obtained from the U 20 national team; all these players scored 100 points. At the time of the FASH collection, the U 20 players stayed in a 3-day training camp to prepare for a friendly match. It is expected that these young footballers are only invited to play in such an event, when they are free of pain with respect to their hamstrings and have unlimited physical function. Additionally and due to their higher trainings volume, these national team players can be expected to be in a better physical performance when compared with the players who are active in lower leagues.

Test–retest reliability was demonstrated during a 2–3-day interval in all 140 participants in the initial FASH validation [4] and in the present investigation. We recommend further longitudinal research to clarify if the FASH scores in a football population without hamstring injuries differ during the course of a season as a result from the different loadings in training and match.

### Limitations and future perspectives

Our FASH-G analyses were based on football players from different German leagues. As a control group, field hockey players were also evaluated. Taking into account, however, that the Greek FASH version was validated in track and field athletes, we strongly feel that the results can be generalised for all sports. Our hamstring-injured group was not homogeneous with respect to the time interval from the injury to the evaluation, and no formal staging was performed. Furthermore, longitudinal data from the injured group were not collected. This could enable better planning of rehabilitation and return to sport and has to be done in future FASH analyses. Finally, further research is needed in terms of translation and cross-cultural adaptation of this condition-specific questionnaire in other languages to promote comparative international studies.

## Conclusions

The FASH-G was demonstrated to be an effective instrument to evaluate German footballers with acute hamstring injuries. We recommend the FASH questionnaire to quantify a patient’s clinical severity, longitudinally document the effectiveness of a treatment, compare different patient populations, and facilitate comparative research in different countries.

## Abbreviations

FASH: Functional Assessment Scale for Acute Hamstring Injuries
FASH-G: Functional Assessment Scale for Acute Hamstring Injuries—German version
ICC: Intra‐class correlation coefficient
MDC: Minimal detectable change
Q: Question
SD: Standard deviation
SEM: Standard error of measurement
